# Development and Utilization of Introgression Lines Using Synthetic Octaploid Wheat (*Aegilops tauschii* × Hexaploid Wheat) as Donor

**DOI:** 10.3389/fpls.2018.01113

**Published:** 2018-08-03

**Authors:** Dale Zhang, Yun Zhou, Xinpeng Zhao, Linlin Lv, Cancan Zhang, Junhua Li, Guiling Sun, Suoping Li, Chunpeng Song

**Affiliations:** ^1^Institute of Plant Stress Biology, State Key Laboratory of Cotton Biology, School of Life Sciences, Henan University, Kaifeng, China; ^2^School of Life Sciences, Henan Normal University, Xinxiang, China

**Keywords:** wheat, *Aegilops tauschii*, quantitative trait loci, agronomic traits, introgression lines

## Abstract

As the diploid progenitor of common wheat, *Aegilops tauschii* Cosson (DD, 2*n* = 2x = 14) is considered to be a promising genetic resource for the improvement of common wheat. In this work, we demonstrated that the efficiency of transferring *A. tauschii* segments to common wheat was clearly improved through the use of synthetic octaploid wheat (AABBDDDD, 2*n* = 8x = 56) as a “bridge.” The synthetic octaploid was obtained by chromosome doubling of hybrid F_1_ (*A. tauschii* T015 × common wheat Zhoumai 18). A set of introgression lines (BC_1_F_8_) containing 6016 *A. tauschii* segments was developed and displayed significant phenotype variance among lines. Twelve agronomic traits, including growth duration, panicle traits, grain traits, and plant height (PH), were evaluated. And transgressive segregation was identified in partial lines. Additionally, better agronomic traits could be observed in some lines, compared to the recurrent parent Zhoumai 18. To verify that the significant variance of those agronomic traits was supposedly controlled by *A. tauschii* segments, 14 quantitative trait loci (QTLs) for three important agronomic traits (thousand kernel weight, spike length, and PH) were further located in the two environments (Huixian and Zhongmou), indicating the introgression of favorable alleles from *A. tauschii* into common wheat. This study provides an ameliorated strategy to improve common wheat utilizing a single *A. tauschii* genome.

## Introduction

Wheat (*Triticum aestivum* L.) is one of the most important cereal crops, accounting for 20% of the calories consumed by humans (Brenchley et al., 2012). Based on hybridization among varieties, many wheat varieties have now been bred through modern cultivation procedures and it should be noted that the process of wheat breeding has been greatly accelerated by the utilization of core collection in China. However, the genetic background of wheat varieties is becoming increasingly consistent, due to their derivation from only a few core collections (Tian et al., 2005; Hao et al., 2006; Xiao et al., 2012), which is currently leading to an increasingly severe risk of abiotic and biotic stress. It has long been realized that the exploration and utilization of desirable genes from wild relatives is an effective approach to improving the genetic background of common wheat (Fu and Somers, 2009; Nevo, 2014). To date, this strategy has been used to transfer many alien genes/QTLs from wild relatives into fine cultivars, and 1BL/1RS is regarded as the most successful alien introgression in wheat-breeding programs (Lukaszewski, 1990, 2000; Jiang et al., 1993; Ren et al., 2009; Gill et al., 2011; Qi et al., 2011). The 1RS arm in translocation lines could not only compensate for the loss of the relevant wheat arms 1BS, but also confer positive heterotic effect to grain yield. In addition, many other wild relatives, including 6VS of *Dasypyrum villosum* (Chen et al., 2013), 2S of *Aegilops speltoides* (Klindworth et al., 2012), 7Ag of *Thinopyrum ponticum* (Niu et al., 2014), and 6P of *Agropyron cristatum* (Luan et al., 2010; Ye et al., 2015; Zhang et al., 2015), have also been further utilized for the improvement of common wheat.

*Aegilops tauschii* Cosson (DD, 2*n* = 2x = 14) is an annual, self-pollinated plant with a high level of genetic variability for disease resistance, productivity traits, and abiotic stress resistance (Singh et al., 2012). It is naturally distributed in central Eurasia, spreading from northern Syria and Turkey to western China. In China, it is mainly distributed in the Yili area of Xinjiang and the middle reaches of the Yellow River (including Shanxi and Henan provinces; Wei et al., 2008). Concerning its genetic background, *A. tauschii* can be subdivided into two phylogenetic lineages, designated as L1 and L2, which are broadly affiliated with *A. tauschii* ssp. *tauschii* and *A. tauschii* ssp. *strangulata*, respectively (Dvorak et al., 1998; Mizuno et al., 2010; Wang et al., 2013). Most of the exploited *A. tauschii* is generally derived from Transcaucasus and northern Iran, since it is believed that the *A. tauschii* in these regions (mainly from the L2 lineage) is involved in the origin of wheat D genome (Wang et al., 2013). By contrast, little is known about the genetic and phenotypic characteristics of *A. tauschii* (mainly L1 lineage) from the eastern and southern populations (i.e., those from Syria, Afghanistan, Pakistan, Central Asia, and China) (Matsuoka et al., 2009). Owing to the long genetic distance between L1 and L2, it is therefore believed that the genetic variation type of *A. tauschii* (L1 lineage) is more abundant than that of the wheat D genome (Lubbers et al., 1991; Dvorak et al., 1998, 2012; Wang et al., 2013). Therefore, like many wild crop progenitors, *A. tauschii* is considered to be a promising gene donor for the improvement of common wheat (Kilian et al., 2011).

As the diploid progenitor of common wheat, it is convenient to transfer *A. tauschii* genes into common wheat via recombination between homologous chromosomes. In addition, it is also possible that undesirable gene linkages can be easily broken by repeated backcrossing with common wheat (Gill and Raupp, 1987). To date, synthetic hexaploid wheat (tetraploid wheat × *A. tauschii*) has mainly been exploited as a “bridge” for transferring some superior genes of *A. tauschii* into common wheat (Miranda et al., 2007). Many previous researchers have identified and located numerous QTLs from synthetic hexaploid wheat with some of the QTLs being located on the D genome through advanced backcross population or introgression lines (ILs; Pestsova et al., 2006; Kunert et al., 2007; Naz et al., 2008; Yu et al., 2014). In addition, the desirable traits of *A. tauschii* may also be transferred to common wheat through direct crossing. Gill and Raupp (1987) proposed the first systematic direct gene transfer protocol. Wheat genomes A, B, and D could be improved concurrently through the hybridization of synthetic hexaploid wheat with common wheat. In comparison, unique advantages have been found in the hybridization of *A. tauschii* with common wheat, because this provides a strategy to transfer desired D genome regions (carrying target alleles) without disrupting adaptive allelic combinations (located in the A and B genomes). However, this method has drawn little attention (Fritz et al., 1995; Cox et al., 2006; Olson et al., 2013) due to the high sterility in the hybrid F_1_ generation, caused by distant hybridization and extremely low ripening rates resulting from the backcross of the hybrid F_1_ with the recurrent parent.

Fortunately, the above-mentioned challenge could be overcome through the use of the synthetic octaploid wheat (AABBDDDD, 2*n* = 8x = 56), obtained by chromosome doubling of hybrid F_1_ (*A. tauschii* × hexaploid wheat), although this has seldom been reported in the literature. In addition, *A. tauschii* from the same region has been generally regarded as more suitable for hybridization with common wheat, compared to strains from other areas, due to its broad ecological adaptation to the native area (Matsuoka et al., 2009). In this work, a series of ILs (BC_1_F_8_) was developed through the media of synthetic octaploid wheat, obtained by direct crossing of common wheat and *A. tauschii* from the same region in China. Various agronomic traits of these ILs were extensively investigated and analyzed. In addition, 14 major QTLs for three important agronomic traits, which were derived from *A. tauschii*, were successfully identified in the two environments.

## Materials and Methods

### Plant Materials

The diploid *A. tauschii* ssp. *tauschii* accession T015 (2*n* = 14, DD) was originally derived from Henan province. Zhoumai 18 (2*n* = 42, AABBDD), a type of control variety of cultivar registered in Henan province, was applied as the recurrent parent in this study.

### Production of F_1_ Hybrids Between Common Wheat and *A. tauschii*

Based on the traditional breeding method, *A. tauschii* accession T015 and Zhoumai 18 were directly crossed and the hybrid F_1_ seeds were taken away 16 days after pollination. The method of embryo removal was reported by Sirkka and Immonen (1993). Seeds were surface sterilized for 8 min with 0.1% HgCl_2_ and rinsed three times in 20 mL ddH_2_0. All handling of seeds and embryos was undertaken under sterile conditions in a laminar flow hood. Embryos were removed from the seeds and transferred to the endosperm of barley; the barley embryos were removed and the scutellums of the hybrid embryos were put in their place. An embryo culture media was used containing a mixture of 4.1 g/L Murashige and Skoog salts (Murashige and Skoog, 1962) with 3% sucrose and no hormone at pH 5.8. The hybrid embryos were incubated in darkness at 25°C for 2 weeks and developed etiolated seedlings with roots, and then the hybrid seedlings were cultivated at 21°C in a 16 h photoperiod (50 μmol/m^2^⋅s^1^, fluorescent light) over the summer.

### Chromosome Doubling Treatment and Population Construction

The method of chromosome doubling was reported by Taira et al. (1991). The hybrid F_1_ seedlings were transferred to the greenhouse in September and were grown for 8 weeks at 21 ± 4°C with 10 h of supplemental light. The F_1_ plantlets with well-formed tillers were uprooted from the soil and divided into two parts. One part was replanted as a control without treatment, and the other part was washed in running water. The roots of each plant were then cut back to a 4–5 cm length and immersed in beakers containing a 0.05% (*w/v*) colchicine solution of pH 7.0, supplemented with a 1.5% (*v/v*) solution of dimethyl sulfoxide (DMSO). Treatments were conducted for a 16 h period at room temperature. After the treatment, the roots were thoroughly washed in running water for 24 h. All the plants were transplanted into a greenhouse until flowering and seed formation.

**FIGURE 1:**
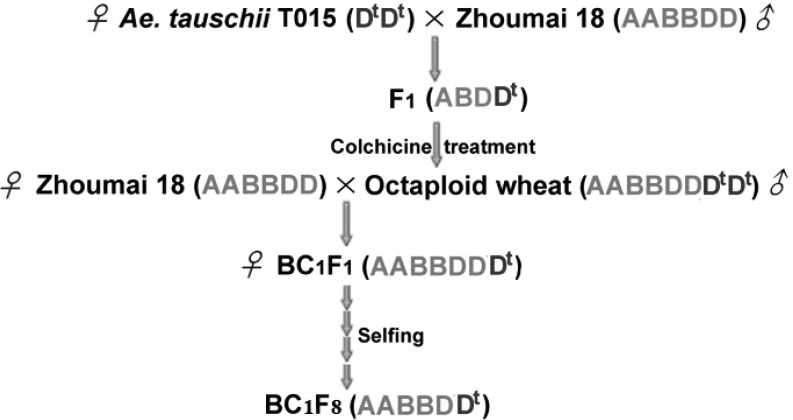
Development of introgression lines by transferring chromosome segments from *A. tauschii* T015 into the wheat cultivar Zhoumai 18. D^t^ highlighted in blue designates the genome of *A. tauschii*.

The following year, emasculated florets of Zhoumai 18 were pollinated by synthetic octaploid wheat to produce 10 BC_1_F_1_ seeds. Afterward, the entire BC_1_F_1_ seeds were cultivated and self-fertilized to acquire BC_1_F_2_ generation. About 400 seeds of BC_1_F_2_ were randomly selected followed by further successive self-fertilization for six times to generate a BC_1_F_8_ population (**Figure 1**), in which 379 plants were randomly selected for genotyping and phenotyping in the present study. This population and Zhoumai 18 were cultivated in the 2015–2016 crop season, on the wheat breeding farms of the Huixian and Zhongmou, respectively. Seeds were sown at a distance of 10 cm between plants, and a 30 cm gap between rows, and were grown under consistent field conditions. The recurrent parent Zhoumai 18 was planted as a control.

### Chromosome Karyotype and FISH of Synthetic Octaploid Wheat

The seeds of synthetic octaploid wheat were germinated at 25°C for 2–3 days. About 2 cm long root tips were treated for karyotyping chromosome preparation. Chromosome preparation and FISH were performed according to the method described by Andres and Kuraparthy (2013). The synthetic oligonucleotides pAs-1 and pSc119.2-1 were marked by 6-carboxytetramethylrhodamine (Tamra) and Alexa Fluor-488-dUTP, respectively (Tang et al., 2014). For sample examination, a drop of pre-mixed DAPI solution (Sangon Biotech, Shanghai, China) was deposited on each slide, and chromosomes were observed by an Olympus BX63 fluorescence microscope (Olympus Corporation, Tokyo, Japan).

### Investigation of Agronomic Traits

Twelve agronomic traits, including days to heading (DH), days to flowering (DF), plant height (PH), spike length (SL), spikelets (SPI), spikelet density (SD), grain number main spike (GNS), thousand kernel weight (TKW), grain length (GL), grain width (GW), grain perimeter (GP), and grain length/grain width (GL/GW), were scored by the method described in Li and Li (2006). PH was recorded just before harvest. DH and DF were noted in the field. After harvest, GNS, SL, and SPI were determined from three main spikes per line, while TGW, GL, GW, and GP were determined from three to five plants.

### Map Construction and QTL Analysis

DNA was extracted from the fresh leaves of ILs and Zhoumai 18 in 2014 using the method described by Olson et al. (2013). The genetic map was constructed based on the physical positions of simple sequence repeat (SSR) markers from wheat D genome^1^. PCR reactions for SSR were performed using the method described by Röder et al. (1998). SSR markers were anchored and grouped into the seven *A. tauschii* chromosomes through sequence alignment between the primers and reference genome (AL8/78 accession; Zhao et al., 2017). The calculation of segment lengths and genome ratios followed the method described by Liu et al. (2006). The QTLs for agronomic traits were identified using QTL IciMapping Ver 4.0 (Meng et al., 2015). RSTEP-LRT-ADD mapping (stepwise regression-based likelihood ratio test for additive QTL) was adopted and a significant threshold of likelihood of odds (LOD) was estimated by running 1000 permutations with a type I error of 0.05.

### Statistical Analysis

All statistical analyses were performed on IBM^®^ statistics 19 (SPSS Inc.), including frequency distribution, correlation coefficient (Pearson correlation), and analysis of variance (ANOVA). ANOVA-general linear model (GLM) was performed to determine the significance of differences between the genotypes of the lines and environments. Genotype-by-environment (G × E) interactions were also analyzed using ANOVA-GLM.

## Results

### Development of Introgression Lines Through Synthetic Octaploid Wheat

**FIGURE 2:**
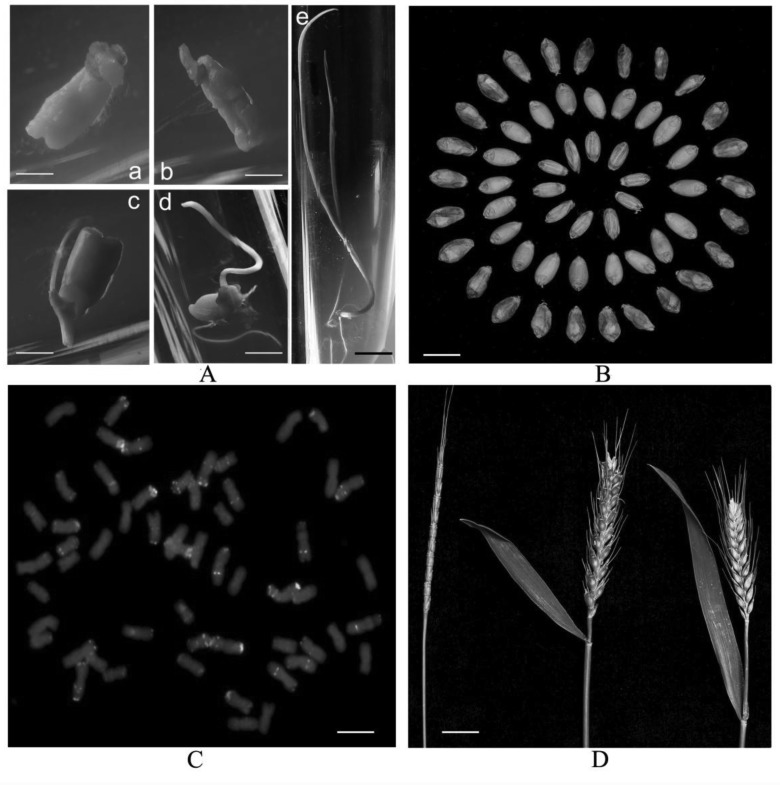
The phenotype characteristics in the process of the development of hybrid F_1_ and synthetic octaploid wheat. **(A)** Embryo culture, (a) to culture 2 days in darkness. Scale bars = 3 mm; (b) to culture 4 days in darkness. Scale bars = 3 mm; (c) to culture 8 days in darkness. Scale bars = 3 mm; (d) to culture 12 days in darkness. Scale bars = 3 mm; and (e) the hybrid seedling in light treatment. Scale bars = 2 cm. **(B)** Seeds of synthetic octaploid wheat and parents, outer cycle: seeds of synthetic octaploid wheat; middle cycle: Zhoumai 18; inner cycle: *A. tauschii* T015. Scale bars = 7 mm. **(C)** chromosome karyotype of synthetic octaploid wheat. Scale bars = 10 μm. **(D)** Spike characteristic of synthetic octaploid wheat and parents, left: *A. tauschii* T015; right: Zhoumai 18. Scale bars = 3 cm.

The ripening rates of reciprocal crosses exhibited significant differences utilizing *A. tauschii* T015 and Zhoumai 18 as parents (**Table 1**). Altogether 73 caryopses were obtained by pollinating 118 emasculated florets of *A. tauschii* T015, with a ripening rate of 61.9%. In contrast, no caryopses were obtained by pollinating 212 emasculated florets of Zhoumai 18. Caryopses collected 16 days after pollination were dissected, and not all of them were found to contain normal embryos (well-developed primordium and scutellum), and about 37.0% contained embryos. Moreover, the embryos were always found floating in a watery endosperm. The normal embryos on the endosperm of barley could germinate and grow into seedlings (**Figure 2A**). Some of the normally developed seedlings were backcrossed with Zhoumai 18 as the female parent, without obtaining any seed. The other seedlings were treated via colchicine to generate amphidiploid seeds (**Figure 2B**). Though these seeds were not full, they could grow normally, exhibiting a chromosome number of 56 in their root tip cells (**Figure 2C**). Except for the prominent characteristics of *A. tauschii* in glume color and hardness, the developed synthetic octaploid wheat showed an analogous phenotype with its male parent (**Figure 2D**). In total, 10 BC_1_F_1_ seeds were obtained through pollinating 16 emasculated florets of Zhoumai 18 with synthetic octaploid wheat as the male parent. Afterward, these BC_1_F_1_ plants successively self-fertilized for eight generations to generate 379 ILs (BC_1_F_8_), in which their phenotypic traits were stabilized after several generations, with no phenotype segregation found in each line, implying the cytogenetical stability of these lines. Furthermore, the chromosome karyotypes of the root tip cell were observed in four selected lines with good agricultural traits, and the number of chromosome in each line was determined to be 42 (Supplementary Figure S1).

**Table 1:** Crossing/backcrossing outcomes for *A. tauschii*/SOW × *T. aestivum*.

	Cross patterns			
	T015 × Zhoumai18	Zhoumai18 × T015	Backcross of hybrid F1 with Zhoumai18 (aaa)	Backcross of SOW with Zhoumai18 (bbb)
No. of florets pollinated	118	212	224	16
No. of caryopses formed	73	0	–	–
No. of embryos formed	27	0	–	–
No. of crossed seeds formed	–	–	0	10

*SOW, synthetic octaploid wheat.*

### Numbers and Positions of Introgressed *A. tauschii* Segments

**FIGURE 3:**
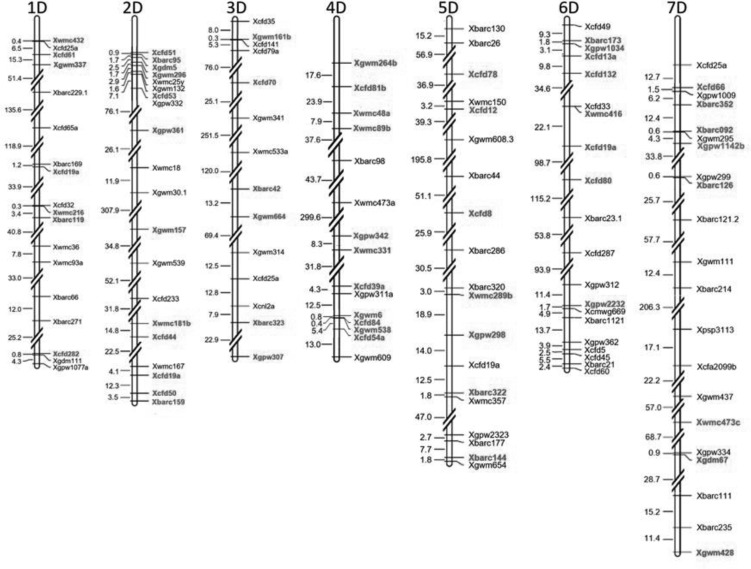
Physical map constructed based on the 127 polymorphic SSR markers between parents. Polymorphic markers in the advanced backcross population are highlighted in red. The unit of distance is megabasepairs (Mb).

To identify the distribution of chromosome segments from *A. tauschii* in the wheat D genome, 379 BC_1_F_8_ lines were successfully genotyped using SSR markers. Altogether 261 SSR markers were selected to construct a genetic map from the GrainGene 2.0 database. Polymorphism was detected in 130 SSR markers between *A. tauschii* T015 and Zhoumai 18, and 62 of these were established to be polymorphic in ILs, accounting for 47.7%. The numbers of polymorphic markers on each chromosome were found rather even, with an average value of 8.9 per chromosome. Excluding three unidentified markers, a physical map was constructed based on the 127 polymorphic SSR markers between parents, which displayed heterogeneous distribution on seven linkage groups of D genome, with a total length of 3954.48 Mb (**Figure 3**). The physical map illustrates that these polymorphic markers in different chromosomes, or different chromosome regions, exhibit uneven distribution. For example, some markers are concentrated in the same region with a minimum gap of only 0.11 Mb. However, huge distances were also found for some other markers. For instance, the distance between *Xgwm157* and *Xgwm30.1* on chromosome 2D was determined to be 307.9 Mb.

Since each line may contain more than one chromosome segment, altogether 6016 segments from *A. tauschii* were determined in ILs. Specifically, these ILs contained 5120 homozygous and 896 heterozygous segment (Supplementary Table S4), with an average of 13.51 homozygous and 2.37 heterozygous segments in each line. The number of segments ranged from 1 to 25 in each line, and only a single introgressed segment was observed in one line. Using the physical positions of the SSR markers, the size of each introgressed segment, the number of unique segment, and the ratios accounting for the whole donor genome were estimated (**Table 2**). The sizes of the introgressed segments ranged from 1.3 to 238.9 Mb, with an average size of 33.45 Mb in homozygous and 31.46 Mb in heterozygous segments. In addition, the distribution of chromosome segments from *A. tauschii* exhibited clear differences in the wheat D genome, and *A. tauschii* segments in each line were counted and graphed in Supplementary Table S1 and Supplementary Figure S2. Typically, the introgression fragments from 1D of *A. tauschii* showed the least 651 fragments, only accounting for 10.8%, and those from 4D of *A. tauschii* possessed the most 1086 fragments, accounting for 18.5%. These results clearly reveal that the chromosome segments of *A. tauschii* have been transferred into common wheat by the “bridge” of synthetic octaploid wheat, which effectively broadens the genetic basis of common wheat.

**Table 2:** The size of introgressed segments detected in the ILs and cumulative proportion in the donor genome.

Chr.			Homozygous segments		Heterozygous segments		Maximum chromosome coverage (%)
	Polymorphic markers	Unique segments	No. of segments	Average length (Mb)	No. of segments	Average length (Mb)	
1D	7	9	981	17.53	105	12.48	20.44
2D	12	20	560	19.87	502	12.14	47.55
3D	7	11	665	42.16	49	27.89	30.38
4D	11	21	914	58.64	65	71.92	55.52
5D	8	8	733	20.11	64	28.62	27.91
6D	9	16	619	59.86	32	50.26	48.05
7D	8	9	648	15.98	79	16.93	23.75
Total	62	94	5120	33.45	896	31.46	36.23


### Phenotypic Variation of Introgression Lines

**FIGURE 4:**
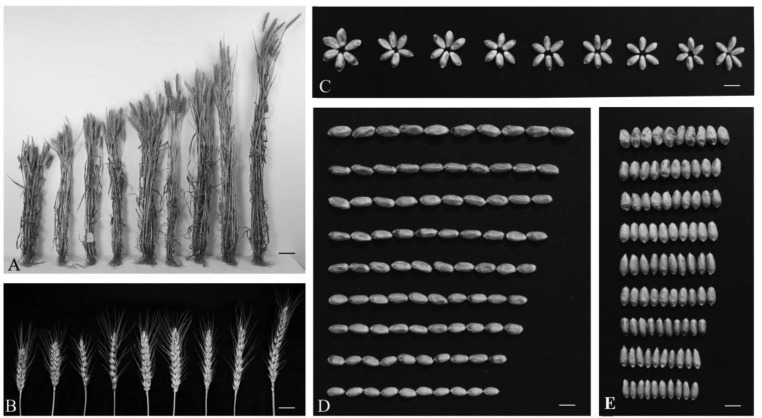
The various phenotype traits from the introgression lines. **(A)** Plant height of partial strains. Scale bars = 8 cm. **(B)** Spike length of partial strains. Scale bars = 2 cm. **(C)** Thousand kernel weight of partial strains. Scale bars = 5 mm. **(D)** Grain length of partial strains. Scale bars = 5 mm. **(E)** Grain width of partial strains. Scale bars = 5 mm.

Some typical traits of *A. tauschii* could be observed in partial lines of ILs. For instance, the glume of some lines exhibited enhanced hardness and deepened color. Consequently, owing to the hardened glume, the spike threshing became difficult with enhanced pre-harvest sprouting resistance. As listed in **Table 3**, significant differences in many agronomic traits could be found among lines, including growth duration, panicle traits, grain traits, and PH (**Figure 4**). In addition, some lines showed apparent transgressive segregation. All the phenotype frequencies were normally distributed in the Huixian and Zhongmou environments (Supplementary Figures S3, S4), demonstrating a skewness range of -0.18∼0.72. PH showed the highest degree of variation in the ILs. The ranges of variation of PH in Huixian and Zhongmou were found to be 53.60–118.63 and 46.65–113.45 cm, with SD values of 11.76 and 11.89, respectively. TKW demonstrated the highest degree of variation in ILs, compared with other grain traits, and many lines with prominently increased TKW values appeared. For Huixian and Zhongmou, 34 and 24 lines presented more than 10% increased TKW than Zhoumai 18, respectively. The panicle traits, mainly consisting of SL, SPI, SD, and GNS, also exhibited significant differences among ILs, with the highest degree of variation found for the GNS. In the Huixian and Zhongmou observations, the variation regions of GNS were determined to be 32.30–73.50 and 34.75–78.00, respectively, with SD values of 6.39 and 7.29, respectively.

**Table 3:** Twelve agronomic traits measured from the recurrent parents and the introgression lines in Huixian and Zhongmou.

Traits	Location	Parent	Introgression lines					
		Zhoumai 18	Mean	SD	Min–Max	C.V.(%)	Skewness	Kurtosis
DH	ZM	195.00	197.37	2.50	191.00-206.33	1.26	0.12	0.04
	HX	187.56	188.20	1.78	180.00-194.00	0.95	-0.15	1.32
DF	ZM	197.88	200.89	2.27	195.75-208.50	1.13	0.22	-0.12
	HX	192.72	193.94	1.82	189.00-199.00	0.94	0.22	-0.15
SL	ZM	9.27	9.98	1.06	6.95-13.58	10.60	0.15	0.44
	HX	8.47	9.77	0.97	7.10-12.87	9.91	0.26	0.35
SPI	ZM	23.25	21.95	1.25	18.50-26.00	5.70	0.17	0.51
	HX	21.02	21.56	1.20	18.00-25.33	5.55	0.07	0.37
GNS	ZM	59.33	54.40	7.29	34.75-78.00	13.40	0.19	-0.003
	HX	55.33	53.10	6.39	32.30-73.50	12.03	0.09	0.13
SD	ZM	24.73	22.24	2.65	15.84-32.37	11.93	0.54	0.71
	HX	26.14	22.26	2.38	16.26-33.02	10.71	0.54	0.93
PH	ZM	76.55	75.24	11.89	46.65-113.45	15.81	0.40	0.19
	HX	78.86	77.19	11.76	53.60-118.63	15.24	0.72	0.65
TKW	ZM	49.54	47.99	4.42	33.81-60.96	9.22	-0.13	0.005
	HX	48.63	48.27	4.01	37.48-59.02	8.31	0.08	-0.16
GL	ZM	5.96	6.58	0.38	5.51-7.55	5.82	-0.10	-0.21
	HX	5.98	6.44	0.40	5.46-7.41	6.27	-0.14	-0.48
GW	ZM	3.18	3.37	0.20	2.89-3.96	5.96	-0.004	-0.28
	HX	3.27	3.28	0.20	2.80-3.78	5.97	-0.05	-0.75
GP	ZM	15.25	16.85	0.97	14.30-19.53	5.74	-0.16	-0.32
	HX	15.46	16.44	1.03	13.98-18.73	6.29	-0.18	-0.71
GL/GW	ZM	1.89	1.97	0.09	1.71-2.25	4.62	0.11	0.09
	HX	1.84	1.98	0.09	1.70-2.28	4.71	0.19	0.49


DH, day to heading; DF, day to flowering; PH, plant height; SL, spike length; SPI, spikelets; SD, spikelet density; GNS, grain number main spike; TKW, thousand kernel weight; GL, grain length; GW, grain width; GP, grain perimeter; GL/GW, grain length/grain width; ZM, Zhongmou; HX, Huixian.

To detect the factors causing significant changes from the phenotypes described above, an ANOVA analysis of genotype, environment, and their interactions was conducted (**Table 4**). Significant differences between genotypes were found for all 12 traits investigated. The *F*-value ranged from 4.72 for GNS to 117.52 for DH. The environment had a large influence on all 12 traits. In particular, DH and DF were the traits most significantly influenced by environment since the cultivation time was not synchronized between the two environments. Significant differences of G × E interaction were observed for the other 11 traits, except for SD, indicating obvious interaction between genotypes and their environment.

**Table 4:** *F* values of ANOVA-GLM for genotype and environment as well as their interaction in the introgression lines.

Traits	Genotype (G)		Environment (E)		G × E interaction	
	*df*	*F*	*df*	*F*	*df*	*F*
DH	378	117.52**	1	28967.90**	378	21.36**
DF	378	82.00**	1	28120.59**	378	20.77**
PH	378	69.01**	1	148.59**	378	5.22**
SL	378	22.86**	1	132.51**	378	1.77**
SPI	378	6.53**	1	72.11**	378	1.44**
SD	378	14.69**	1	2.26 NS	378	1.02 NS
GNS	378	4.72**	1	16.68**	378	2.76**
GP	378	11.67**	1	338.57**	378	5.30**
GL/GW	378	15.45**	1	537.04**	378	6.05**
GL	378	11.08**	1	188.98**	378	4.87**
GW	378	14.54**	1	20.97**	378	7.15**
TKW	378	36.77**	1	38.03**	378	12.33**


*NS, not significant; ^∗∗^significant difference at *P* < 0.01.*

### Correlation Analysis Among Phenotypic Traits

Genetic correlations were calculated among lines for the agronomic traits in the population (Supplementary Tables S2, S3). In Huixian, the two traits of DH and DF showed significant positive correlation with each other (*r* = 0.860, *p* < 0.01), and were also positively correlated with SL and SPI. Meanwhile, a negative correlation was found between these two traits and PH, TKW, GNS, and SD. Among the panicle traits, SL and SPI displayed a positive correlation (*r* = 0.158, *p* < 0.01), and SD was observed to be negatively correlated with the former trait (*r* = -0.843, *p* < 0.01). As for the grain traits, TKW demonstrated a positive correlation with GL, GW, GP, and GL/GW. Concerning the trait of PH, it was found to be negatively correlated with GNS, SPI, and SD, but positively correlated with SL, TKW, GL, GW, GP, and GL/GW. Observations from the Zhongmou environment showed analogous correlations to those in Huixian, with the exception of a positive correlation between growth duration and GNS (*r* = 0.114, *p* < 0.01), and the negative correlation between TKW and GL/GW.

### QTL Analysis of Partial Agronomic Traits in Introgression Lines

**FIGURE 5:**
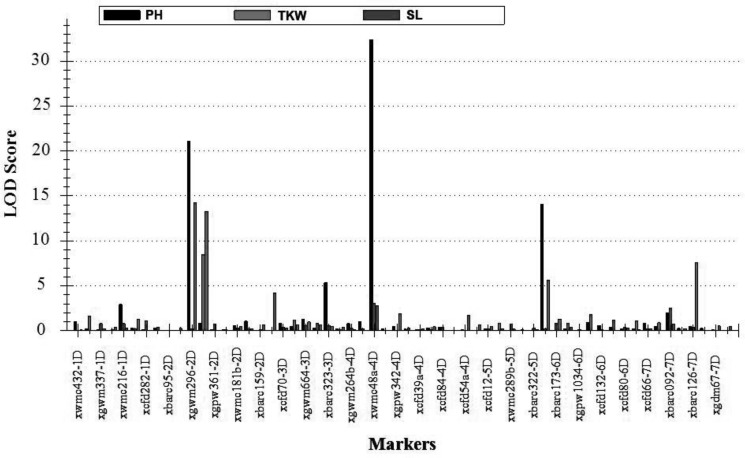
The positions of putative QTLs of three agronomic traits detected in both Huixian and Zhongmou regions. PH, plant height; TKW, thousand kernel weight; SL, spike length.

To elucidate the significant changes in the 12 traits mentioned above, supposedly controlled by *A. tauschii* segments, QTLs for three important agronomic traits (TKW, SL, and PH) of them were further identified (**Table 5**). The TKW is an important factor affecting yield. Three major QTLs for TKW, designated *QTKW.At-2D, QTKW.At-4D*, and *QTKW.At-6D*, were detected on the chromosomes 2D, 4D, and 6D, based on ICIM analysis, respectively (**Figure 5**), and the *QTKW.At-2D* could be detected in both the Huixian and Zhongmou areas. As clearly shown in **Table 5**, the positive alleles of additive effect were derived from *A. tauschii*, further revealing the huge value of genes from *A. tauschii* as a wild wheat resource (Singh et al., 2012). The *QTKW.At-2D* displayed the similar phenotypic variance values (PVEs) of 9.24 and 9.19% in Huixian and Zhongmou, corresponding to the additive effect of the values 1.22 and 1.35 g.

**Table 5:** Analysis of putative QTLs for partial agronomic traits in ILs.

Trait	QTL	Environment	Marker	Position (Mb)	LOD	PVE (%)	Add
TKW	*QTKW.At-2D*	Huixian	*Xcfd53*	2D (26.2)	7.05	9.24	1.22
		Zhongmou			7.02	9.19	1.35
		*Combined*			8.48	10.69	1.28
	*QTKW.At-4D*	Huixian	*Xwmc48a*	4D (71.1)	3.11	3.60	1.37
	*QTKW.At-6D*	Zhongmou	*Xcfd13a*	6D (16.6)	3.12	3.90	-0.88
PH	*QPH.At-2D*	Huixian	*Xgwm296*	2D (20.0)	17.89	12.61	4.25
		Zhongmou			18.61	13.29	4.41
		*Combined*			21.12	13.95	4.33
	*QPH.At-3D*	Huixian	*Xbarc323*	3D (602.1)	3.55	2.60	-2.01
		Zhongmou			5.17	3.82	-2.46
		*Combined*			5.35	3.63	-2.29
	*QPH.At-4D*	Huixian	*Xwmc48a*	4D (71.1)	34.72	27.55	11.09
		Zhongmou			22.37	17.22	8.87
		*Combined*			32.40	23.86	9.99
	*QPH.At-5D*	Huixian	*Xbarc144*	5D (562.8)	15.17	10.75	4.73
		Zhongmou			10.36	6.83	3.73
		*Combined*			14.10	8.92	4.12
	*QPH.At-1D*	Huixian	*Xwmc216*	1D (373.5)	2.66	2.06	1.72
SL	*QSL.At-2D.1*	Huixian	*Xcfd53*	2D (26.2)	14.19	12.88	0.35
		Zhongmou			10.48	8.04	0.30
		*Combined*			13.29	10.46	0.32
	*QSL.At-2D.2*	Huixian	*Xgwm296*	2D (20.0)	7.92	6.08	0.24
		Zhongmou			18.68	13.71	0.40
		*Combined*			14.25	9.81	0.31
	*QSL.At-5D*	Huixian	*Xbarc144*	5D (562.8)	4.49	3.47	0.22
		Zhongmou			5.27	3.59	0.24
		*Combined*			5.63	3.80	0.23
	*QSL.At-7D*	Huixian	*Xbarc126*	7D (91.3)	8.02	6.27	0.25
		Zhongmou			7.22	5.11	0.24
		*Combined*			7.61	5.11	0.22
	*QSL.At-3D*	Zhongmou	*Xgwm161b*	3D (8.1)	4.27	3.33	-0.19
	*QSL.At-4D*	Huixian	*Xgpw342*	4D (451.6)	3.67	3.71	-0.29


LOD, likelihood of odds; PVE, phenotypic variance explained by each QTL; Add, additive effect. Positive values of Add indicate the effects increasing trait values by *A. tauschii* alleles.

Spike length is one of the significant spike traits for the improvement of common wheat. Altogether six major QTLs for SL, designated *QSL.At-2D.1, QSL.At-2D.2, QSL.At-3D, QSL.At-4D, QSL.At-5D*, and *QSL.At-7D* were detected in Huixian and Zhongmou (**Figure 5**), and *QSL.At-2D.1, QSL.At-2D.2, QSL.At-5D*, and *QSL.At-7D* were detected in both locations. *QSL.At-3D* was only detected in Zhongmou, whereas *QSL.At-4D* was observed in Huixian. Among these major QTLs, the PVEs of *QSL.At-2D.1* on chromosome 2D were the highest, and could explain 12.88 and 8.04% of the phenotypic variance in Huixian and Zhongmou corresponding to the additive effect of the values 0.35 and 0.30 cm.

The PH is also an important agronomic trait, and four major QTLs for PH, designated as *QPH.At-2D, QPH.At-3D, QPH.At-4D*, and *QPH.At-5D* hereafter (**Figure 5**), were observed in both Huixian and Zhongmou. The other QTL of *QPH.At-1D* was only detected in Huixian. Among them, the *QPH.At-4D* on chromosome 4D provided the highest explanation for the phenotypic variances in Huixian and Zhongmou, 27.55 and 17.22%, respectively. Moreover, the PVEs of *QPH.At-2D* and *QPH.At-5D* were also relatively high in both places, and could explain 13.95 and 8.92% of the mean phenotypic variance, corresponding to the mean additive effect of the values of 4.33 and 4.12 cm, respectively.

## Discussion

Direct introgression from diploid species into hexaploid wheat has been explored as a possible applied plant-breeding technique for the rapid introgression of useful traits. Gill and Raupp (1987) reported that a total of 219 hybrid embryos were obtained by the hybridization of hexaploid wheat “Wichita” or “Newton” with 3l accessions of *A. squarrosa* (2*n* = 14) as male parent, but only 24 F_1_ hybrids were grown to maturity. Another work of direct crossing between *T. aestivum* and *A. tauschii* was reported by Sehgal et al. (2011). Their results showed that about 51.72% of the pollinated florets produced embryo-carrying caryopses and 6.80 plants for every 100 florets pollinated were obtained when *A. tauschii* was used as the female parent. However, only 0.09 plants for every 100 florets pollinated were obtained in the reciprocal. In this work, about 61.90% of the pollinated florets produced embryo-carrying caryopses, and 22.9% caryopses generated normal embryos with *A. tauschii* as the female parent. No embryo-carrying caryopses were obtained in the reciprocal. These results suggest that the hybrid F_1_ was easily obtained when *A. tauschii* was used as the female parent rather than the male parent. In addition, a major bottleneck in direct gene transfer is the high sterility in the F_1_ from distant hybridization and extremely low ripening rates by backcrosses of hybrid F_1_ with the recurrent parent. In a study by Sehgal et al. (2011), self-seed was hardly expected in the hybrid F_1_ from distant hybridization. Moreover, the untreated tillers produced an average of 0.47 backcross seeds per 100 florets, while the colchicine treated tillers could produce an average of 14.9 backcross seeds per 100 florets pollinated (with a range of 8.33–26.88 seeds). In this work, the backcross of synthetic octaploid wheat as male parent with the recurrent parent Zhoumai 18 resulted in a ripening rate of 62.5%. Therefore, only direct crosses with *A. tauschii* as the male parent were adopted for gene transfer (Cox et al., 2006), and using synthetic octaploid wheat as the male parent could obviously enhance backcross ripening rates with the recurrent parent. Specifically, the hybrid F_1_ was obtained by *A. tauschii* as the female parent and was then doubled to generate the synthetic octaploid wheat. In addition, compared with single gene transfer, the development of ILs can incorporate more than one useful gene simultaneously into common wheat. Liu et al. (2006) cultivated an ILs containing Am3 chromosome segments, which included 162 homozygous and 166 heterozygous segments. In this work, the ILs containing 6016 *A. tauschii* segments were developed using synthetic octaploid wheat as a “bridge,” and no phenotype segregation was found in each line, which indicates that these lines are cytogenetically stable, and could be utilized more easily through further breeding.

It is well known that polyploids are more prone to receive portions of alien chromosomal introgression from related weedy species compared to diploids. Despite their overall inferior agronomic performance, wild and weedy species are likely to contain genetic factors that can increase the yield of modern varieties. In other words, quantitative traits of modern varieties may be improved using wild and weedy species (Frey et al., 1984). The 1RS arm in the translocation line 1BL/1RS wheat, for example, carries a battery of resistance traits and adaptation to abiotic stresses, as well as high-yield traits (Friebe et al., 1996; Sharma et al., 2011). In the process of improving common wheat by utilizing the desirable genes of *A. tauschii*, the yield, kernel weight, protein concentration, and kernel hardness were evaluated, based on 147 BC_2_F_1_-derived families from crossing between elite common wheat lines and *A. tauschii* (Fritz et al., 995). The results indicated that introgression of *A. tauschii* germplasm into the wheat genome had fewer effects on agronomic performance, compared to the extreme phenotypic differences between the two species. Variability for yield and protein was actually lower among strains carrying larger estimated amounts of *A. tauschii* segments. Thus, *A. tauschii* has been deemed to have a relatively neutral impact on the agronomic and quality traits of wheat but to serve as a source of important resistance genes. To date, many resistance genes of *A. tauschii* have been transferred into common wheat through the use of synthetic hexaploid wheat as a “bridge” (Naz et al., 2008; Dunckel et al., 2015; Wang et al., 2016). Through a doubled haploid (DH) population derived from synthetic-derived bread wheat line SYN1 and FHB-susceptible line Ocoroni, Zhu et al. (2016) identified a major QTL of Fusarium head blight (FHB) resistance on chromosome 2D, accounting for 25% of the phenotypic variation explained. Liu et al. (2006) investigated nine agronomic traits of 97 ILs containing Am3 chromosome segments, in which the Am3 was synthesized by the crossing of *Triticum carthlicum* with *A. tauschii*. The phenotype traits from ILs showed obvious change, and some strains displayed better agronomic traits than the recurrent parent. In this work, the agronomic traits among lines also showed significant variation. Although most of the strains were similar to the recurrent parent Zhoumai 18, some of them demonstrated apparent transgressive segregation (**Table 3**). In addition, 14 quantitative trait loci (QTLs) among three important agronomic traits (TKW, SL, and PH) were further located in the Huixian and Zhongmou, confirming the introgression of favorable alleles from *A. tauschii* into common wheat.

Genetic correlations between traits are due to linkage and/or pleiotropy and indicate the magnitude and direction of correlated response to selection, as well as the relative efficiency of indirect selection (Holland, 2006). When traits are highly correlated, plant breeders can select for the trait with higher heritability and simultaneously indirectly select for the other trait. The genetic correlation of agronomic traits of 188 recombinant inbred lines (RILs) from the spring wheat “Louise” × “Penawawa” were analyzed by Carter (2011), who found that flowering date and PH, as well as maturity date and PH, were moderately correlated. PH was positively correlated to grain yield, with taller plants having higher grain yield potential. Kumar et al. (2007) reported that grain yield was significantly correlated to SL in two mapping populations. In this work, PH was found to be negatively correlated with GNS, SPI, and SD, but positively correlated with SL, TKW, GL, GW, GP, and GL/GW. Similarly, TKW and SL showed significant positive correlation.

Plenty of studies have attempted to map QTL for grain yield and yield components of wheat under non-stress conditions (Kato et al., 2000; Börner et al., 2002; Huang et al., 2004, 2006; Mccartney et al., 2005; Marza et al., 2006; Narasimhamoorthy et al., 2006; Kuchel et al., 2007; Kumar et al., 2007; Cuthbert et al., 2008; Heidari et al., 2011). However, it is still necessary to confirm the role of important markers associated with grain yield across different genetic backgrounds and environments. Huang et al. (2003) reported detecting a major *QTgw.ipk-2D* on chromosome 2DL with a boundary from Xgwm539 to Xgdm6 in a BC_2_F_2_ population derived from a cross between the common wheat and the synthetic wheat. This QTL could explain 15.4% of the phenotypic variation. Crossa et al. (2007) used two linear mixed models to assess marker-trait associations. They identified significant associations between grain yield and the DArT markers wPt-4413 on chromosome 2D. Using association mapping, Edae et al. (2014) detected one stable QTL for grain yield on chromosome 2DS, under both irrigated and rain-fed conditions. The QTL associated with the DArT marker *wpt6531* is about 8 cM away from the *wpt4144* marker, which was associated with yield in the study of Crossa et al. (2007). Using two different RILs populations, Kumar et al. (2007) identified one QTL for grain yield on chromosome 2D with a boundary from *X*gwm261 to *Xcdo1379*. In addition, Narasimhamoorthy et al. (2006) detected a QTL for grain yield linked to Xgwm261. Interestingly, according to the linkage map of Crossa et al. (2007), the SSR markers (*Xgwm261*) were linked to the DArT marker wPt-4413, spanning 3.2 cM. Four QTLs for TKW (Huang et al., 2004, 2006; Cuthbert et al., 2008) were identified close (from 1.7 cM for *Xgwm296* to 7.9 cM for *Xwmc601*) to the DArT markerwPt-4413 on chromosome 2D, according to the linkage map of Crossa et al. (2007). Azadi et al. (2015) identified that two QTLs (*QTgw.abrii-2D1* and *QTgw.abrii-2D3*) were also close to the DArT marker wPt-4413. In the present study, one major QTL for TKW, designated *QTKW.At-2D*, was detected on the Xcfd53 of chromosome 2D in the Huixian and Zhongmou environments (**Table 3**). The QTL (*QTKW.At-2D*) was also close to the DArT marker wPt-4413 according to the linkage map of Crossa et al. (2007). Identification of this QTL for grain yield/ TKW at the same position suggests a possible pleiotropic QTL and also indicates that this region may play an important role in improving grain yield. When averaged across two environments, this QTL could explain 10.69% of the phenotypic variation, corresponding to the additive effect values of 1.28. The *Xcfd53* was associated with positive effects on TKW. Typically, the accession 150679, containing the above-mentioned marker, showed TKW values of 59.02 and 60.96 g in the two districts, providing high increments of 22.2% and 24.4% compared with Zhoumai 18, respectively. These results reveal that favorable alleles from *A. tauschii* can improve important agronomic traits of an elite wheat variety, even though *A. tauschii* itself is inferior to the cultivated variety in the phenotypic traits.

## Conclusion

A set of ILs containing only *A. tauschii* segments was established by using synthetic octaploid wheat (AABBDDDD, 2*n* = 8x = 56) as a “bridge.” This bridge was obtained by the chromosome doubling of hybrid F_1_ (*A. tauschii* T015 × common wheat Zhoumai 18). The agronomic traits among lines also showed significant phenotype variation. For every trait, some lines displayed better performance than the recurrent parent. In addition, 14 QTLs for three important agronomic traits (TKW, PH, and SL) were further located in Huixian and Zhongmou regions, respectively.

## Author Contributions

SL and CS conceived and designed the study. DZ, YZ, XZ, LL, CZ, JL, and GS generated the data and performed the analysis. DZ and YZ contributed reagents, materials, and analysis tools. DZ, YZ, SL, and CS wrote and revised the paper. All authors read and approved the final manuscript.

## Conflict of Interest Statement

The authors declare that the research was conducted in the absence of any commercial or financial relationships that could be construed as a potential conflict of interest.

## Footnotes

1 http://wheat.pw.usda.gov/cgi-bin/GG3/

## References

[B1] Andres R. J. Kuraparthy V. (2013). Development of an improved method of mitotic metaphase chromosome preparation compatible for fluorescence in situ hybridization in cotton. *J. Cotton Sci.* 17 149–156.12582918

[B2] Azadi A. Mardi M. Hervan E. M. Mohammadi S. A. Moradi F. Tabatabaee M. T. (2015). QTL Mapping of yield and yield components under normal and salt-stress conditions in bread wheat (*Triticum aestivum* L.). *Plant Mol. Biol. Rep.* 33 102–120. 10.1007/s11105-014-0726-0

[B3] Börner A. Schumann E. Fürste A. Cöster H. Leithold B. Röder S. (2002). Mapping of quantitative trait loci determining agronomic important characters in hexaploid wheat (*Triticum aestivum* L.). *Theor. Appl. Genet.* 105 921–936. 10.1007/s00122-002-0994-1 12582918

[B4] Brenchley R. Spannagl M. Pfeifer M. Barker G. L. D’amore R. Allen A. M. (2012). Analysis of the bread wheat genome using whole-genome shotgun sequencing. *Nature* 491 705–710. 10.1038/nature11650 23192148 PMC3510651

[B5] Carter A. (2011). Genetic mapping of quantitative trait loci associated with important agronomic traits in the spring wheat (*Triticum aestivum* L.) cross ‘Louise’ by ‘Penawawa’. *Crop Sci.* 51 84–95. 10.2135/cropsci2010.03.0185

[B6] Chen P. D. You C. F. Hu Y. Chen S. W. Zhou B. Cao A. Z. (2013). Radiation-induced translocations with reduced *Haynaldia villosa* chromatin at the Pm21 locus for powdery mildew resistance in wheat. *Mol. Breed.* 31 477–484. 10.1007/s11032-012-9804-x

[B7] Cox T. S. Hatchett J. H. Gill B. S. Raupp W. J. Sears R. G. (2006). Agronomic performance of hexaploid wheat lines derived from direct crosses between wheat and *Aegilops squarrosa*. *Plant Breed.* 105 271–277. 10.1111/j.1439-0523.1990.tb01285.x

[B8] Crossa J. Burgueno J. Dreisigacker S. Vargas M. Herrera-Foessel S. A. Lillemo M. (2007). Association analysis of historical bread wheat germplasm using additive genetic covariance of relatives and population structure. *Genetics* 177 1889–1913. 10.1534/genetics.107.078659 17947425 PMC2147943

[B9] Cuthbert J. L. Somers D. J. Brule-Babel A. L. Brown P. D. Crow G. H. (2008). Molecular mapping of quantitative trait loci for yield and yield components in spring wheat (*Triticum aestivum* L.). *Theor. Appl. Genet.* 117 595–608. 10.1007/s00122-008-0804-5 18516583

[B10] Dunckel S. M. Olson E. L. Rouse M. N. Bowden R. L. Poland J. A. (2015). Genetic mapping of race-specific stem rust resistance in the synthetic hexaploid W7984 × Opata M85 mapping population. *Crop Sci.* 55 1–9. 10.2135/cropsci2014.11.0755

[B11] Dvorak J. Deal K. R. Luo M. C. You F. M. Von Borstel K. Dehghani H. (2012). The origin of spelt and free-threshing hexaploid wheat. *J. Hered.* 103 426–441. 10.1093/jhered/esr152 22378960

[B12] Dvorak J. Luo M. C. Yang Z. L. Zhang H. B. (1998). The structure of the *Aegilops tauschii* genepool and the evolution of hexaploid wheat. *Theor. Appl. Genet.* 97 657–670. 10.1007/s001220050942

[B13] Edae E. A. Byrne P. F. Haley S. D. Lopes M. S. Reynolds M. P. (2014). Genome-wide association mapping of yield and yield components of spring wheat under contrasting moisture regimes. *Theor. Appl. Genet.* 127 791–807. 10.1007/s00122-013-2257-8 24408378

[B14] Frey K. J. Cox T. S. Rodgers D. M. Bramel-Cox P. (1984). Increasing cereal yields with genes from wild and weedy species. *Genetics* 4 51–68.

[B15] Friebe B. Jiang J. Raupp W. J. Mcintosh R. A. Gill B. S. (1996). Characterization of wheat-alien translocations conferring resistance to diseases and pests: current status. *Euphytica* 91 59–87. 10.1007/BF00035277

[B16] Fritz A. K. Cox T. S. Gill B. S. Sears R. G. (1995). Molecular marker-facilitated analysis of introgression in winter wheat ×*Triticum tauschii* populations. *Crop Sci.* 35 1691–1695. 10.2135/cropsci1995.0011183X003500060030x

[B17] Fu Y. B. Somers D. J. (2009). Genome-wide reduction of genetic diversity in wheat breeding. *Crop Sci.* 49 161–168. 10.2135/cropsci2008.03.0125

[B18] Gill B. S. Friebe B. R. White F. F. (2011). Alien introgressions represent a rich source of genes for crop improvement. *Proc. Natl. Acad. Sci. U.S.A.* 108 7657–7658. 10.1073/pnas.1104845108 21527718 PMC3093505

[B19] Gill B. S. Raupp W. J. (1987). Direct genetic transfers from *Aegilops squarrosa* L. to hexaploid wheat. *Crop Sci.* 27 445–450. 10.2135/cropsci1987.0011183X002700030004x

[B20] Hao C. Y. Zhang X. Y. Wang L. F. Dong Y. S. Shang X. W. Jia J. Z. (2006). Genetic diversity and core collection evaluations in common wheat germplasm from the northwestern spring wheat region in China. *Mol. Breed.* 17 69–77. 10.1007/s11032-005-2453-6

[B21] Heidari B. Sayed-Tabatabaei B. E. Saeidi G. Kearsey M. Suenaga K. (2011). Mapping QTL for grain yield, yield components, and spike features in a doubled haploid population of bread wheat. *Genome* 54 517–527. 10.1139/g11-017 21635161

[B22] Holland J. B. (2006). Estimating genotypic correlations and their standard errors using multivariate restricted maximum likelihood estimation with SAS Proc MIXED. *Crop Sci.* 46 642–654. 10.2135/cropsci2005.0191

[B23] Huang X. Q. Cloutier S. Lycar L. Radovanovic N. Humphreys D. G. Noll J. S. (2006). Molecular detection of QTLs for agronomic and quality traits in a doubled haploid population derived from two Canadian wheats (*Triticum aestivum* L.). *Theor. Appl. Genet.* 113 753–766. 10.1007/s00122-006-0346-7 16838135

[B24] Huang X. Q. Coster H. Ganal M. W. Roder M. S. (2003). Advanced backcross QTL analysis for the identification of quantitative trait loci alleles from wild relatives of wheat (*Triticum aestivum* L.). *Theor. Appl. Genet.* 106 1379–1389. 10.1007/s00122-002-1179-7 12750781

[B25] Huang X. Q. Kempf H. Ganal M. W. Röder M. S. (2004). Advanced backcross QTL analysis in progenies derived from a cross between a German elite winter wheat variety and a synthetic wheat (*Triticum aestivum* L.). *Theor. Appl. Genet.* 109 933–943. 10.1007/s00122-004-1708-7 15243706

[B26] Jiang J. M. Friebe B. Gill B. S. (1993). Recent advances in alien gene transfer in wheat. *Euphytica* 73 199–212. 10.1007/BF0003670023321705

[B27] Kato K. Miura H. Sawada S. (2000). Mapping QTLs controlling grain yield and its components on chromosome 5A of wheat. *Theor. Appl. Genet.* 101 1114–1121. 10.1007/s001220051587

[B28] Kilian B. Mammen K. Millet E. Sharma R. Graner A. Salamini F. (2011). “Aegilops,” in *Wild Crop Relatives: Genomic and Breeding Resources. Cereals*, ed. Kole C. (Berlin: Springer), 1–76.

[B29] Klindworth D. L. Niu Z. Chao S. Friesen T. L. Jin Y. Faris J. D. (2012). Introgression and characterization of a goatgrass gene for a high level of resistance to ug99 stem rust in tetraploid wheat. *G3* 2 665–673. 10.1534/g3.112.002386 22690376 PMC3362296

[B30] Kuchel H. Williams K. J. Langridge P. Eagles H. A. Jefferies S. P. (2007). Genetic dissection of grain yield in bread wheat. I. QTL analysis. *Theor. Appl. Genet.* 115 1029–1041. 10.1007/s00122-007-0629-7 17713755

[B31] Kumar N. Kulwal P. L. Balyan H. S. Gupta P. K. (2007). QTL mapping for yield and yield contributing traits in two mapping populations of bread wheat. *Mol. Breed.* 19 163–177. 10.1007/s11032-006-9056-8

[B32] Kunert A. Naz A. A. Dedeck O. Pillen K. Léon J. (2007). AB-QTL analysis in winter wheat: I. Synthetic hexaploid wheat (*T. turgidum* ssp. dicoccoides × *T. tauschii*) as a source of favourable alleles for milling and baking quality traits. *Theor. Appl. Genet.* 115 683–695. 10.1007/s00122-007-0600-7 17634917

[B33] Li L. H. Li X. Q. (2006). *Wheat Germplasm Resource Description Specification and Data Standard.* Beijing: China Agriculture Press, 59–61.

[B34] Liu S. B. Zhou R. G. Dong Y. C. Li P. Jia J. Z. (2006). Development, utilization of introgression lines using a synthetic wheat as donor. *Theor. Appl. Genet.* 112 1360–1373. 10.1007/s00122-006-0238-x 16550399

[B35] Luan Y. Wang X. G. Liu W. H. Li C. Y. Zhang J. P. Gao A. N. (2010). Production and identification of wheat-*Agropyron cristatum* 6P translocation lines. *Planta* 232 501–510. 10.1007/s00425-010-1187-9 20490543

[B36] Lubbers E. L. Gill K. S. Cox T. S. Gill B. S. (1991). Variation of molecular markers among geographically diverse accessions. *Genome* 34 354–361. 10.1139/g91-057

[B37] Lukaszewski A. J. (1990). Frequency of 1RS.1AL and 1RS.1BL translocations in United States wheats. *Crop Sci.* 30 1151–1153. 10.2135/cropsci1990.0011183X003000050041x

[B38] Lukaszewski A. J. (2000). Manipulation of the 1RS.1BL translocation in wheat by induced homoeologous recombination. *Crop Sci.* 40 216–225. 10.2135/cropsci2000.401216x

[B39] Marza F. Bai G. H. Carver B. F. Zhou W. C. (2006). Quantitative trait loci for yield and related traits in the wheat population Ning7840 × Clark. *Theor. Appl. Genet.* 112 688–698. 10.1007/s00122-005-0172-3 16369760

[B40] Matsuoka Y. Nishioka E. Kawahara T. Takumi S. (2009). Genealogical analysis of subspecies divergence and spikelet-shape diversification in central Eurasian wild wheat *Aegilops tauschii* Coss. *Plant Syst. Evol.* 279 233–244. 10.1007/s00606-009-0159-7

[B41] Mccartney C. A. Somers D. J. Humphreys D. G. Lukow O. Ames N. Noll J. (2005). Mapping quantitative trait loci controlling agronomic traits in the spring wheat cross RL4452 × ’AC Domain’. *Genome* 48 870–883. 10.1139/g05-055 16391693

[B42] Meng L. Li H. H. Zhang L. Y. Wang J. K. (2015). QTL IciMapping: integrated software for genetic linkage map construction and quantitative trait locus mapping in biparental populations. *Crop J.* 3 269–283. 10.1016/j.cj.2015.01.001

[B43] Miranda L. M. Murphy J. P. Marshall D. Cowger C. Leath S. (2007). Chromosomal location of Pm35, a novel *Aegilops tauschii* derived powdery mildew resistance gene introgressed into common wheat (*Triticum aestivum* L.). *Theor. Appl. Genet.* 114 1451–1456. 10.1007/s00122-007-0530-4 17356863

[B44] Mizuno N. Yamasaki M. Matsuoka Y. Kawahara T. Takumi S. (2010). Population structure of wild wheat D-genome progenitor *Aegilops tauschii* Coss: implications for intraspecific lineage diversification and evolution of common wheat. *Mol. Ecol.* 19 999–1013. 10.1111/j.1365-294X.2010.04537.x 20149088

[B45] Murashige T. Skoog F. (1962). A revised medium for rapid growth and bio assays with tobacco tissue cultures. *Physiol. Plant.* 15 473–497. 10.1111/j.1399-3054.1962.tb08052.x

[B46] Narasimhamoorthy B. Gill B. S. Fritz A. K. Nelson J. C. Brown-Guedira G. L. (2006). Advanced backcross QTL analysis of a hard winter wheat x synthetic wheat population. *Theor. Appl. Genet.* 112 787–796. 10.1007/s00122-005-0159-0 16463062

[B47] Naz A. A. Kunert A. Lind V. Pillen K. Léon J. (2008). AB-QTL analysis in winter wheat: II. Genetic analysis of seedling and field resistance against leaf rust in a wheat advanced backcross population. *Theor. Appl. Genet.* 116 1095–1104. 10.1007/s00122-008-0738-y 18338154 PMC2358941

[B48] Nevo E. (2014). Evolution of wild emmer wheat and crop improvement. *J. Syst. Evol.* 52 673–696. 10.1111/jse.12124

[B49] Niu Z. Klindworth D. L. Yu G. Friesen T. L. Chao S. Jin Y. (2014). Development and characterization of wheat lines carrying stem rust resistance gene Sr43 derived from *Thinopyrum ponticum*. *Theor. Appl. Genet.* 127 969–980. 10.1007/s00122-014-2272-4 24504553

[B50] Olson E. L. Rouse M. N. Pumphrey M. O. Bowden R. L. Gill B. S. Poland J. A. (2013). Simultaneous transfer, introgression, and genomic localization of genes for resistance to stem rust race TTKSK (Ug99) from *Aegilops tauschii* to wheat. *Theor. Appl. Genet.* 126 1179–1188. 10.1007/s00122-013-2045-5 23377571

[B51] Pestsova E. G. Borner A. Roder M. S. (2006). Development and QTL assessment of *Triticum aestivum*-*Aegilops tauschii* introgression lines. *Theor. Appl. Genet.* 112 634–647. 10.1007/s00122-005-0166-1 16341683

[B52] Qi L. L. Pumphrey M. O. Friebe B. Zhang P. Qian C. Bowden R. L. (2011). A novel Robertsonian translocation event leads to transfer of a stem rust resistance gene (Sr52) effective against race Ug99 from *Dasypyrum villosum* into bread wheat. *Theor. Appl. Genet.* 123 159–167. 10.1007/s00122-011-1574-z 21437597

[B53] Ren T. H. Yang Z. J. Yan B. J. Zhang H. Q. Fu S. L. Ren Z. L. (2009). Development and characterization of a new 1BL.1RS translocation line with resistance to stripe rust and powdery mildew of wheat. *Euphytica* 169 207–213. 10.1007/s10681-009-9924-5

[B54] Röder M. S. Korzun V. Wendehake K. Plaschke J. Tixier M. Leroy P. (1998). A microsatellite map of wheat. *Genetics* 149 2007–2023. 10.1016/B0-12-227620-5/00113-0 9691054 PMC1460256

[B55] Sehgal S. K. Kaur S. Gupta S. Sharma A. Kaur R. Bains N. S. (2011). A direct hybridization approach to gene transfer from *Aegilops tauschii* Coss. To *Triticum aestivum* L. *Plant Breed.* 130 98–100. 10.1111/j.1439-0523.2010.01817.x

[B56] Sharma S. Xu S. Z. Ehdaie B. Hoops A. Close T. J. Lukaszewski A. J. (2011). Dissection of QTL effects for root traits using a chromosome arm-specific mapping population in bread wheat. *Theor. Appl. Genet.* 122 759–769. 10.1007/s00122-010-1484-5 21153397 PMC3037480

[B57] Singh S. Chahal G. S. Singh P. K. Gill B. S. (2012). Discovery of desirable genes in the germplasm pool of *Aegilops tauschii* Coss. *Indian J. Genet. Plant Breed.* 72 271–277.

[B58] Sirkka A. Immonen T. (1993). Comparison of callus culture with embryo culture at different times of embryo rescue for primary triticale production. *Euphytica* 70 185–190. 10.1007/BF00023758

[B59] Taira T. Shao Z. Z. Hamawaki H. Larter E. N. (1991). The effect of colchicine as a chromosome doubling agent for wheat-rye hybrids as influenced by pH, method of application, and post-treatment environment. *Plant Breed.* 106 329–333. 10.1111/j.1439-0523.1991.tb00518.x

[B60] Tang Z. Yang Z. Fu S. (2014). Oligonucleotides replacing the roles of repetitive sequences pAs1, pSc119.2, pTa-535, pTa71, CCS1, and pAWRC.1 for FISH analysis. *J. Appl. Genet.* 55 313–318. 10.1007/s13353-014-0215-z 24782110

[B61] Tian Q. Z. Zhou R. H. Jia J. Z. (2005). Genetic diversity trend of common wheat (*Triticum aestivum* L.) in China revealed with AFLP markers. *Genet. Resour. Crop Evol.* 52 325–331. 10.1007/s10722-005-5716-5

[B62] Wang J. Luo M. C. Chen Z. You F. M. Wei Y. Zheng Y. (2013). *Aegilops tauschii* single nucleotide polymorphisms shed light on the origins of wheat D-genome genetic diversity and pinpoint the geographic origin of hexaploid wheat. *New Phytol.* 198 925–937. 10.1111/nph.12164 23374069

[B63] Wang Y. J. Wang C. Y. Quan W. Jia X. J. Fu Y. Zhang H. (2016). Identification and mapping of PmSE5785, a new recessive powdery mildew resistance locus, in synthetic hexaploid wheat. *Euphytica* 207 619–626. 10.1007/s10681-015-1560-7

[B64] Wei H. T. Li J. Peng Z. S. Lu B. R. Zhao Z. J. Yang W. Y. (2008). Relationships of *Aegilops tauschii* revealed by DNA fingerprints: the evidence for agriculture exchange between China and the West. *Prog. Nat. Sci.* 18 1525–1531. 10.1016/j.pnsc.2008.05.022

[B65] Xiao Y. G. Qian Z. G. Wu K. Liu J. J. Xia X. C. Ji W. Q. (2012). Genetic gains in grain yield and physiological traits of winter wheat in Shandong Province, China, from 1969 to 2006. *Crop Sci.* 52 44–56. 10.2135/cropsci2011.05.0246

[B66] Ye X. L. Lu Y. Q. Liu W. H. Chen G. Y. Han H. M. Zhang J. P. (2015). The effects of chromosome 6P on fertile tiller number of wheat as revealed in wheat-*Agropyron cristatum* chromosome 5A/6P translocation lines. *Theor. Appl. Genet.* 128 797–811. 10.1007/s00122-015-2466-4 25656149

[B67] Yu M. Chen G. Y. Zhang L. Q. Liu Y. X. Liu D. C. Wang J. R. (2014). QTL mapping for important agronomic traits in synthetic hexaploid wheat derived from *Aegiliops tauschii* ssp. tauschii. *J. Integr. Agric.* 13 1835–1844. 10.1016/s2095-3119(13)60655-3

[B68] Zhang J. Zhang J. P. Liu W. H. Han H. M. Lu Y. Q. Yang X. M. (2015). Introgression of *Agropyron cristatum* 6P chromosome segment into common wheat for enhanced thousand-grain weight and spike length. *Theor. Appl. Genet.* 128 1827–1837. 10.1007/s00122-015-2550-9 26093609

[B69] Zhao G. Zou C. Li K. Wang K. Li T. Gao L. (2017). The *Aegilops tauschii* genome reveals multiple impacts of transposons. *Nat. Plants* 3 946–955. 10.1038/s41477-017-0067-8 29158546

[B70] Zhu Z. W. Bonnett D. Ellis M. He X. Y. Heslot N. Dreisigacker S. (2016). Characterization of Fusarium head blight resistance in a CIMMYT synthetic-derived bread wheat line. *Euphytica* 208 367–375. 10.1007/s10681-015-1612-z

