# Supplementary material for: Development and Utilization of Introgression Lines Using Synthetic Octaploid Wheat (Aegilops tauschii × Hexaploid Wheat) as Donor
Source: Front Plant Sci. 2018 Aug 3;9:1113. doi: 10.3389/fpls.2018.01113 (PMC6085485; doi:10.3389/fpls.2018.01113)
Supplement: Supplementary file 1 [file Table_1.docx]

**Supplementary Material**

**Development and Utilization of Introgression Lines using Synthetic Octaploid Wheat (*Aegilops tauschii* × Hexaploid Wheat) as Donor**

Dale Zhang^1+^, Yun Zhou^1+^, Xinpeng Zhao^1^, Linlin Lv^1^, Cancan Zhang^1^, Junhua Li^2^, Guiling Sun^1^, Suoping Li^^[[1]](#footnote-1)^^^*^, Chunpeng Song^1*^

**Table S1** Number of introgressed segments in the BC_1_F_8_ population.

| Lines | Homozygous segments | Heterozygous segments | Total |
| --- | --- | --- | --- |
| 150675 | 20 | 1 | 21 |
| 151946 | 14 | 1 | 15 |
| 151741 | 19 | 4 | 23 |
| 150646 | 13 | 0 | 13 |
| 150651 | 7 | 1 | 8 |
| 150686 | 10 | 1 | 11 |
| 150682 | 18 | 2 | 20 |
| 150695 | 13 | 1 | 14 |
| 150639 | 10 | 1 | 11 |
| 150643 | 7 | 2 | 9 |
| 150644 | 4 | 2 | 6 |
| 150678 | 7 | 1 | 8 |
| 150689 | 22 | 1 | 23 |
| 150683 | 20 | 0 | 19 |
| 150672 | 20 | 1 | 20 |
| 150684 | 16 | 1 | 17 |
| 150646 | 7 | 2 | 9 |
| 150640 | 7 | 0 | 7 |
| 150676 | 9 | 7 | 16 |
| 150690 | 15 | 5 | 20 |
| 150679 | 14 | 5 | 19 |
| 150652 | 12 | 5 | 17 |
| 150662 | 17 | 3 | 20 |
| 150673 | 11 | 2 | 13 |
| 150685 | 17 | 2 | 19 |
| 150686 | 14 | 6 | 20 |
| 150687 | 20 | 2 | 21 |
| 150677 | 18 | 1 | 19 |
| 150697 | 21 | 1 | 22 |
| 150644 | 5 | 2 | 7 |
| 150680 | 12 | 4 | 16 |
| 150734 | 16 | 2 | 18 |
| 150760 | 13 | 6 | 18 |
| 150763 | 3 | 0 | 3 |
| 150785 | 17 | 1 | 18 |
| 150793 | 15 | 2 | 16 |
| 150717 | 13 | 2 | 15 |
| 150797 | 19 | 2 | 21 |
| 150722 | 15 | 3 | 18 |
| 150727 | 21 | 1 | 22 |
| 150726 | 13 | 1 | 14 |
| 150741 | 17 | 2 | 19 |
| 150757 | 12 | 5 | 17 |
| 150751 | 16 | 3 | 19 |
| 150713 | 22 | 0 | 22 |
| 150705 | 19 | 3 | 22 |
| 150750 | 18 | 0 | 18 |
| 150742 | 22 | 1 | 23 |
| 150715 | 21 | 1 | 22 |
| 150735 | 16 | 1 | 17 |
| 150755 | 14 | 2 | 16 |
| 150710 | 16 | 3 | 19 |
| 150721 | 12 | 1 | 13 |
| 150787 | 13 | 1 | 14 |
| 150740 | 16 | 1 | 17 |
| 150702 | 20 | 1 | 21 |
| 150718 | 15 | 4 | 19 |
| 150703 | 11 | 5 | 16 |
| 150739 | 15 | 4 | 19 |
| 150773 | 14 | 2 | 16 |
| 150767 | 20 | 0 | 20 |
| 150733 | 13 | 3 | 16 |
| 150737 | 14 | 3 | 17 |
| 150796 | 15 | 4 | 19 |
| 150723 | 11 | 4 | 15 |
| 150731 | 21 | 3 | 23 |
| 150781 | 11 | 1 | 12 |
| 150724 | 16 | 4 | 20 |
| 150738 | 11 | 5 | 16 |
| 150736 | 17 | 2 | 19 |
| 150725 | 11 | 3 | 14 |
| 150749 | 14 | 2 | 16 |
| 150763 | 2 | 1 | 3 |
| 150761 | 10 | 2 | 12 |
| 150762 | 7 | 0 | 7 |
| 150714 | 18 | 3 | 21 |
| 151567 | 17 | 4 | 21 |
| 151585 | 11 | 4 | 15 |
| 151566 | 13 | 4 | 17 |
| 151568 | 12 | 4 | 16 |
| 151569 | 18 | 4 | 22 |
| 151576 | 15 | 3 | 17 |
| 151577 | 8 | 7 | 15 |
| 151579 | 20 | 1 | 21 |
| 151580 | 18 | 2 | 20 |
| 151582 | 13 | 4 | 17 |
| 151583 | 15 | 3 | 18 |
| 151587 | 14 | 4 | 18 |
| 151589 | 13 | 3 | 16 |
| 151590 | 15 | 5 | 20 |
| 151591 | 14 | 2 | 16 |
| 151592 | 14 | 2 | 16 |
| 151593 | 14 | 1 | 15 |
| 151594 | 14 | 1 | 15 |
| 151597 | 9 | 1 | 10 |
| 151599 | 10 | 2 | 12 |
| 151586 | 12 | 2 | 14 |
| 151587 | 3 | 0 | 3 |
| 151571 | 3 | 0 | 3 |
| 151572 | 13 | 4 | 17 |
| 151601 | 9 | 2 | 11 |
| 151603 | 21 | 3 | 24 |
| 151605 | 12 | 1 | 13 |
| 151606 | 13 | 3 | 16 |
| 151609 | 10 | 4 | 14 |
| 151611 | 10 | 8 | 18 |
| 151612 | 18 | 2 | 20 |
| 151613 | 16 | 2 | 18 |
| 151615 | 12 | 3 | 15 |
| 151616 | 9 | 3 | 12 |
| 151618 | 20 | 1 | 21 |
| 151620 | 16 | 0 | 16 |
| 151621 | 17 | 6 | 23 |
| 151622 | 17 | 3 | 20 |
| 151623 | 13 | 1 | 14 |
| 151625 | 18 | 2 | 20 |
| 151626 | 9 | 6 | 15 |
| 151627 | 13 | 1 | 14 |
| 151629 | 12 | 0 | 12 |
| 151630 | 15 | 3 | 18 |
| 151631 | 10 | 4 | 14 |
| 151637 | 16 | 1 | 17 |
| 151640 | 12 | 7 | 19 |
| 151641 | 9 | 1 | 10 |
| 151644 | 14 | 6 | 20 |
| 151648 | 13 | 0 | 13 |
| 151653 | 10 | 0 | 10 |
| 151654 | 20 | 0 | 20 |
| 151656 | 13 | 2 | 15 |
| 151658 | 13 | 2 | 15 |
| 151659 | 19 | 1 | 20 |
| 151661 | 17 | 0 | 17 |
| 151662 | 17 | 1 | 18 |
| 151664 | 13 | 2 | 15 |
| 151665 | 3 | 2 | 5 |
| 151667 | 16 | 2 | 17 |
| 151669 | 11 | 2 | 13 |
| 151674 | 15 | 1 | 16 |
| 151676 | 12 | 4 | 16 |
| 151681 | 18 | 3 | 21 |
| 151688 | 13 | 6 | 19 |
| 151693 | 21 | 0 | 21 |
| 151694 | 16 | 4 | 20 |
| 151696 | 14 | 1 | 15 |
| 151698 | 13 | 1 | 14 |
| 151700 | 9 | 6 | 15 |
| 151702 | 14 | 3 | 17 |
| 151704 | 11 | 7 | 18 |
| 151708 | 9 | 3 | 12 |
| 151722 | 12 | 4 | 16 |
| 151723 | 16 | 3 | 19 |
| 151724 | 14 | 1 | 15 |
| 151725 | 18 | 1 | 19 |
| 151729 | 12 | 4 | 16 |
| 151730 | 15 | 3 | 18 |
| 151731 | 14 | 5 | 19 |
| 151732 | 15 | 0 | 15 |
| 151733 | 12 | 3 | 15 |
| 151735 | 18 | 2 | 20 |
| 151737 | 13 | 5 | 18 |
| 151743 | 7 | 3 | 10 |
| 151744 | 13 | 2 | 15 |
| 151745 | 6 | 4 | 10 |
| 151746 | 3 | 1 | 4 |
| 151747 | 12 | 0 | 12 |
| 151748 | 19 | 1 | 20 |
| 151749 | 16 | 6 | 22 |
| 151753 | 16 | 2 | 18 |
| 151757 | 17 | 4 | 21 |
| 151759 | 18 | 0 | 18 |
| 151760 | 18 | 0 | 18 |
| 151761 | 11 | 0 | 11 |
| 151762 | 15 | 2 | 17 |
| 151765 | 11 | 5 | 16 |
| 151768 | 12 | 0 | 12 |
| 151770 | 9 | 0 | 9 |
| 151773 | 22 | 0 | 22 |
| 151775 | 11 | 1 | 12 |
| 151777 | 15 | 1 | 16 |
| 151779 | 11 | 1 | 12 |
| 151780 | 15 | 4 | 19 |
| 151783 | 18 | 2 | 20 |
| 151785 | 16 | 0 | 16 |
| 151790 | 11 | 0 | 11 |
| 151791 | 12 | 0 | 12 |
| 151792 | 11 | 7 | 18 |
| 151794 | 11 | 4 | 15 |
| 151797 | 21 | 1 | 22 |
| 151798 | 14 | 3 | 17 |
| 151805 | 19 | 3 | 22 |
| 151806 | 19 | 1 | 20 |
| 151808 | 12 | 3 | 15 |
| 151811 | 15 | 1 | 16 |
| 151814 | 18 | 3 | 21 |
| 151816 | 19 | 3 | 22 |
| 151818 | 12 | 4 | 16 |
| 151820 | 12 | 1 | 13 |
| 151822 | 15 | 0 | 15 |
| 151823 | 16 | 2 | 18 |
| 151824 | 10 | 3 | 13 |
| 151825 | 16 | 2 | 18 |
| 151826 | 13 | 2 | 15 |
| 151827 | 13 | 5 | 18 |
| 151828 | 12 | 3 | 15 |
| 151837 | 18 | 2 | 20 |
| 151839 | 11 | 2 | 13 |
| 151841 | 15 | 0 | 15 |
| 151847 | 15 | 2 | 17 |
| 151850 | 24 | 1 | 25 |
| 151852 | 18 | 1 | 19 |
| 151857 | 18 | 1 | 19 |
| 151860 | 11 | 1 | 12 |
| 151862 | 11 | 5 | 16 |
| 151864 | 14 | 2 | 16 |
| 151866 | 18 | 1 | 19 |
| 151867 | 14 | 1 | 15 |
| 151869 | 14 | 5 | 19 |
| 151870 | 16 | 3 | 19 |
| 151872 | 13 | 3 | 16 |
| 151875 | 14 | 3 | 17 |
| 151876 | 9 | 3 | 12 |
| 151880 | 11 | 4 | 15 |
| 151881 | 16 | 2 | 18 |
| 151882 | 16 | 2 | 18 |
| 151885 | 18 | 0 | 18 |
| 151889 | 13 | 3 | 16 |
| 151890 | 12 | 7 | 19 |
| 151891 | 11 | 6 | 17 |
| 151892 | 10 | 2 | 12 |
| 151900 | 8 | 1 | 9 |
| 151901 | 1 | 0 | 1 |
| 151905 | 14 | 2 | 16 |
| 151908 | 13 | 1 | 14 |
| 151910 | 6 | 4 | 10 |
| 151911 | 13 | 4 | 17 |
| 151913 | 15 | 5 | 20 |
| 151915 | 19 | 0 | 19 |
| 151916 | 18 | 0 | 18 |
| 151917 | 15 | 3 | 18 |
| 151918 | 10 | 4 | 14 |
| 151919 | 10 | 3 | 13 |
| 151921 | 14 | 1 | 15 |
| 151924 | 18 | 3 | 21 |
| 151928 | 12 | 0 | 12 |
| 151929 | 11 | 1 | 12 |
| 151930 | 10 | 0 | 10 |
| 151934 | 17 | 1 | 18 |
| 151939 | 11 | 4 | 15 |
| 151940 | 13 | 1 | 14 |
| 151942 | 16 | 0 | 16 |
| 151944 | 10 | 1 | 11 |
| 151947 | 15 | 1 | 16 |
| 151949 | 21 | 2 | 23 |
| 151952 | 17 | 5 | 22 |
| 151954 | 11 | 4 | 15 |
| 151955 | 14 | 1 | 15 |
| 151956 | 14 | 3 | 17 |
| 151960 | 13 | 0 | 13 |
| 151961 | 17 | 1 | 18 |
| 151963 | 8 | 4 | 12 |
| 151965 | 21 | 1 | 22 |
| 151966 | 14 | 4 | 18 |
| 151967 | 14 | 3 | 17 |
| 151968 | 15 | 3 | 18 |
| 151969 | 13 | 3 | 16 |
| 151971 | 3 | 0 | 3 |
| 151972 | 9 | 3 | 12 |
| 151973 | 13 | 2 | 15 |
| 151975 | 8 | 3 | 11 |
| 151977 | 11 | 4 | 15 |
| 151978 | 2 | 0 | 2 |
| 151979 | 12 | 2 | 14 |
| 151982 | 20 | 3 | 23 |
| 151984 | 13 | 4 | 17 |
| 151985 | 11 | 5 | 16 |
| 151986 | 11 | 6 | 17 |
| 151988 | 9 | 5 | 14 |
| 151989 | 7 | 4 | 11 |
| 151993 | 13 | 3 | 16 |
| 151995 | 4 | 0 | 4 |
| 151999 | 18 | 4 | 22 |
| 152001 | 3 | 0 | 3 |
| 152002 | 10 | 2 | 12 |
| 152011 | 16 | 3 | 19 |
| 152012 | 11 | 3 | 14 |
| 152013 | 14 | 2 | 16 |
| 152015 | 13 | 2 | 15 |
| 152022 | 14 | 1 | 15 |
| 152023 | 11 | 2 | 13 |
| 152024 | 15 | 6 | 21 |
| 152025 | 20 | 4 | 24 |
| 152026 | 20 | 5 | 25 |
| 152027 | 18 | 3 | 21 |
| 152028 | 18 | 1 | 19 |
| 152030 | 20 | 3 | 23 |
| 152031 | 22 | 2 | 24 |
| 152033 | 23 | 1 | 24 |
| 152034 | 19 | 4 | 23 |
| 152040 | 22 | 1 | 23 |
| 152041 | 19 | 3 | 22 |
| 152042 | 20 | 3 | 23 |
| 152043 | 22 | 2 | 24 |
| 152044 | 21 | 2 | 23 |
| 152045 | 13 | 0 | 13 |
| 152046 | 7 | 1 | 8 |
| 152047 | 13 | 2 | 15 |
| 152048 | 13 | 2 | 15 |
| 152049 | 12 | 2 | 14 |
| 152050 | 7 | 2 | 9 |
| 152051 | 9 | 6 | 15 |
| 152052 | 11 | 1 | 12 |
| 152055 | 14 | 3 | 17 |
| 152056 | 8 | 1 | 9 |
| 152057 | 16 | 2 | 18 |
| 152058 | 12 | 3 | 15 |
| 152059 | 12 | 1 | 13 |
| 152060 | 11 | 1 | 12 |
| 152061 | 14 | 3 | 17 |
| 152062 | 12 | 6 | 18 |
| 152063 | 16 | 1 | 17 |
| 152064 | 16 | 2 | 18 |
| 152065 | 13 | 2 | 15 |
| 152066 | 12 | 5 | 17 |
| 152067 | 14 | 2 | 16 |
| 152068 | 12 | 1 | 13 |
| 152069 | 14 | 0 | 14 |
| 152070 | 14 | 2 | 16 |
| 152071 | 15 | 1 | 16 |
| 152072 | 13 | 1 | 14 |
| 152073 | 12 | 2 | 14 |
| 152074 | 14 | 2 | 16 |
| 152075 | 11 | 5 | 16 |
| 152076 | 14 | 1 | 15 |
| 152077 | 12 | 3 | 15 |
| 152078 | 7 | 3 | 10 |
| 152079 | 10 | 3 | 13 |
| 152080 | 11 | 2 | 13 |
| 152081 | 7 | 4 | 11 |
| 152082 | 7 | 3 | 10 |
| 152083 | 9 | 3 | 12 |
| 152084 | 9 | 2 | 11 |
| 152085 | 3 | 0 | 3 |
| 152090 | 12 | 5 | 17 |
| 152091 | 11 | 3 | 14 |
| 152092 | 11 | 2 | 13 |
| 152093 | 9 | 4 | 13 |
| 152094 | 9 | 4 | 13 |
| 152095 | 12 | 3 | 15 |
| 152096 | 10 | 1 | 11 |
| 152097 | 13 | 2 | 15 |
| 152098 | 13 | 2 | 15 |
| 152100 | 15 | 1 | 16 |
| 152101 | 13 | 2 | 15 |
| 152102 | 14 | 3 | 17 |
| 152103 | 14 | 1 | 15 |
| 152104 | 12 | 3 | 15 |
| 152110 | 9 | 2 | 11 |
| 152111 | 12 | 3 | 15 |
| 152112 | 9 | 2 | 11 |
| 152113 | 12 | 3 | 15 |
| 152114 | 9 | 3 | 12 |
| 152115 | 11 | 2 | 13 |
| 152116 | 8 | 0 | 8 |
| 152120 | 9 | 2 | 11 |
| 152122 | 9 | 3 | 12 |
| 152123 | 10 | 3 | 13 |
| 152124 | 9 | 4 | 13 |
| 152125 | 10 | 4 | 14 |
| 152126 | 17 | 0 | 17 |
| 152130 | 2 | 0 | 2 |
| 152131 | 13 | 2 | 15 |
| 152132 | 20 | 1 | 21 |
| 15140 | 22 | 2 | 24 |
| 152141 | 18 | 2 | 20 |
| 152145 | 14 | 6 | 20 |
| 152146 | 17 | 1 | 18 |
| 152147 | 18 | 3 | 21 |
| 152148 | 17 | 2 | 19 |
| 152149 | 18 | 4 | 22 |
| Total | 5120 | 896 | 6016 |

Table S2 Correlation coefficients among agronomic traits of ILs in Huixian.

| Traits | DH | DF | SL | SPI | PH | GNS | TKW | GL | GW | GP | GL/GW | SD |
| --- | --- | --- | --- | --- | --- | --- | --- | --- | --- | --- | --- | --- |
| DH | 1.00 |  |  |  |  |  |  |  |  |  |  |  |
| DF | 0.860** | 1.00 |  |  |  |  |  |  |  |  |  |  |
| SL | 0.204** | 0.268** | 1.00 |  |  |  |  |  |  |  |  |  |
| SPI | 0.103* | 0.182** | 0.158** | 1.00 |  |  |  |  |  |  |  |  |
| PH | -0.094 | -0.050 | 0.457** | -0.188** | 1.00 |  |  |  |  |  |  |  |
| GNS | -0.121* | -0.106* | 0.045 | 0.459** | -0.087 | 1.00 |  |  |  |  |  |  |
| TKW | -0.138** | -0.157** | 0.286** | -0.211** | 0.474** | -0.164** | 1.00 |  |  |  |  |  |
| GL | 0.039 | 0.033 | 0.260** | -0.112* | 0.245** | -0.152** | 0.423** | 1.00 |  |  |  |  |
| GW | -0.107* | -0.143** | -0.033 | -0.077 | 0.097 | -0.046 | 0.445** | 0.709** | 1.00 |  |  |  |
| GP | 0.001 | -0.013 | 0.189** | -0.112* | 0.216** | -0.120* | 0.437** | 0.975** | 0.838** | 1.00 |  |  |
| GL/GW | 0.069 | 0.131* | 0.230** | -0.076 | 0.217** | -0.082 | 0.023 | 0.303** | -0.157** | 0.188** | 1.00 |  |
| SD | -0.131* | -0.147** | -0.843** | 0.380** | -0.520** | 0.183** | -0.370** | -0.307** | -0.008 | -0.240** | -0.260** | 1.00 |

Note: DH: Day to heading, DF: Day to flowering, PH: plant height, SL: spike length, SPI: spikelets, SD: spikelet density, GNS: grain number main spike, TKW: thousand kernel weight, GL: grain length, GW: grain width, GP: grain perimete, GL/GW: grain length/grain width.

*: correlation is significant at the 0.05 level (2-tailed), **: correlation is significant at the 0.01 level (2-tailed).

Table S3 Correlation coefficients among agronomic traits of ILs in Zhongmou.

| Traits | DH | DF | SL | SPI | PH | GNS | TKW | GL | GW | GP | GL/GW | SD |
| --- | --- | --- | --- | --- | --- | --- | --- | --- | --- | --- | --- | --- |
| DH | 1.00 |  |  |  |  |  |  |  |  |  |  |  |
| DF | 0.905** | 1.00 |  |  |  |  |  |  |  |  |  |  |
| SL | 0.238** | 0.283** | 1.00 |  |  |  |  |  |  |  |  |  |
| SPI | 0.069 | 0.108* | 0.065 | 1.00 |  |  |  |  |  |  |  |  |
| PH | 0.009 | 0.043 | 0.449** | -0.217** | 1.00 |  |  |  |  |  |  |  |
| GNS | 0.114* | 0.108* | 0.158** | 0.362** | -0.054 | 1.00 |  |  |  |  |  |  |
| TKW | -0.055 | -0.109* | 0.269** | -0.282** | 0.381** | -0.074 | 1.00 |  |  |  |  |  |
| GL | -0.109* | -0.126* | 0.305** | -0.150** | 0.232** | -0.086 | 0.420** | 1.00 |  |  |  |  |
| GW | -0.083 | -0.095 | 0.098 | -0.207** | 0.144** | -0.128* | 0.451** | 0.683** | 1.00 |  |  |  |
| GP | -0.097 | -0.114* | 0.267** | -0.190** | 0.229** | -0.108* | 0.444** | 0.970** | 0.833** | 1.00 |  |  |
| GL/GW | -0.019 | -0.024 | 0.256** | 0.070 | 0.087 | 0.054 | -0.070 | 0.375** | -0.418** | 0.147** | 1.00 |  |
| SD | -0.164** | -0.191** | -0.867** | 0.417** | -0.505** | 0.020 | -0.364** | -0.338** | -0.174** | -0.321** | -0.204** | 1.00 |

Note: DH: Day to heading, DF: Day to flowering, PH: plant height, SL: spike length, SPI: spikelets, SD: spikelet density, GNS: grain number main spike, TKW: thousand kernel weight, GL: grain length, GW: grain width, GP: grain perimete, GL/GW: grain length/grain width.

*: correlation is significant at the 0.05 level (2-tailed), **: correlation is significant at the 0.01 level (2-tailed).


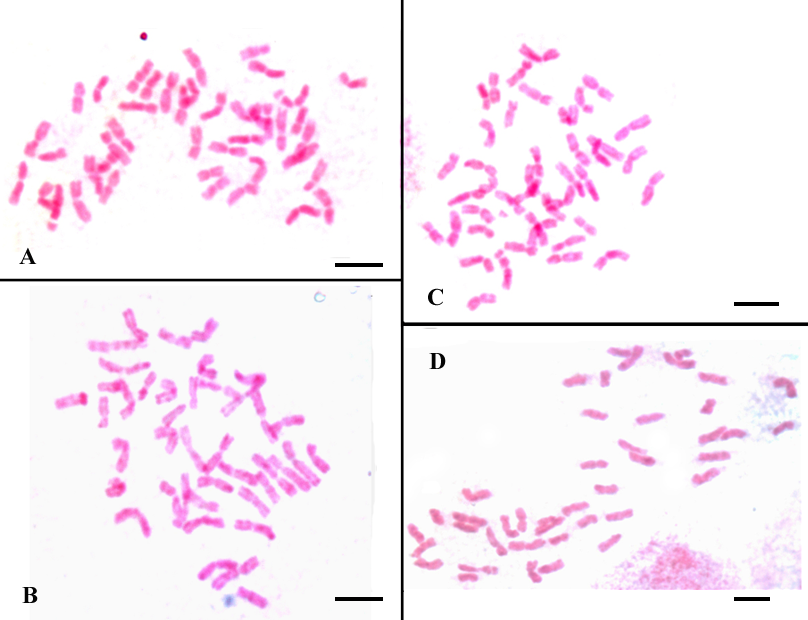


Fig. S1 Chromosome karyotypes of partial introgression lines, Scale bar = 10 µm. A: line 150679; B: line 150689; C: line 151748; D: line 150639

**
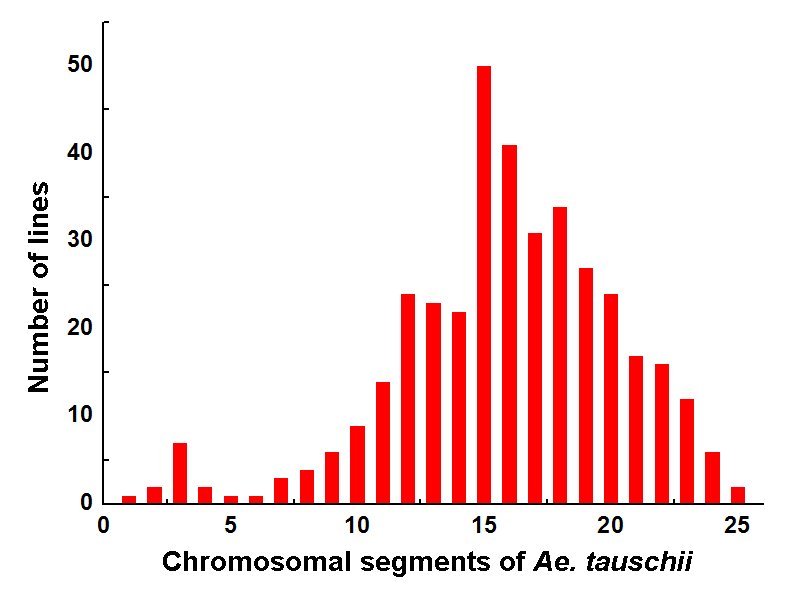
**

**Fig.S2** Distribution of chromosome segments from *Ae. tauschii* accession T015 among the 379 lines.


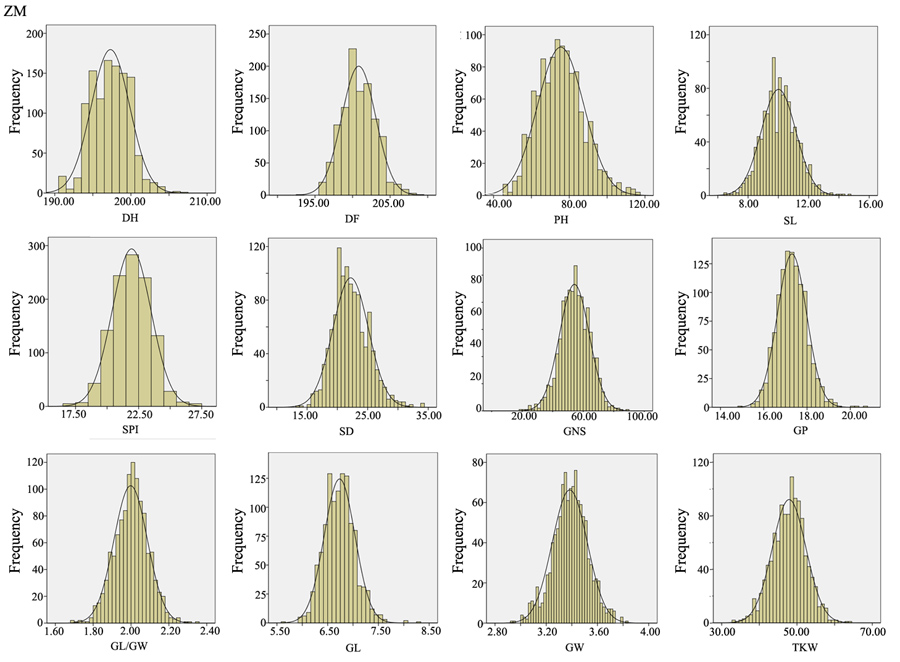


Fig. S3 Frequency distributions of 12 agronomic traits in the introgression lines under Zhongmou environment.


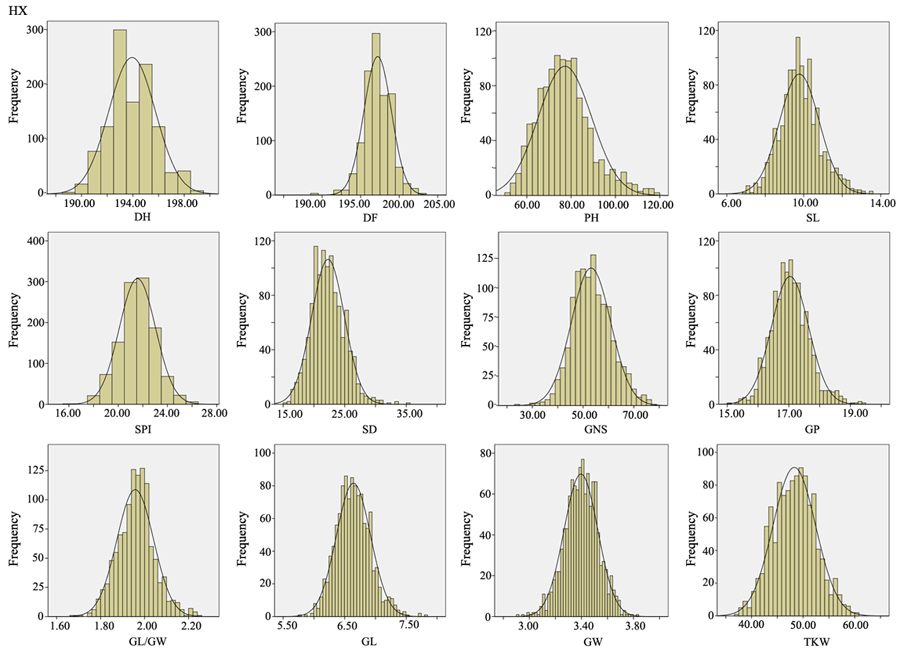


Fig. S4 Frequency distributions of 12 agronomic traits in the introgression lines under Huixian environment.

1. 1Institute of Plant Stress Biology, State Key Laboratory of Cotton Biology, School of Life Science, Henan University, Kaifeng 475004, China

   ^2^ School of Life Sciences, Henan Normal University, Xinxiang 453007, China.

   + Equal contributors

   * Corresponding authors: [lisuoping@henu.edu.cn](mailto:lisuoping@henu.edu.cn) (Suoping Li), [songcp@henu.edu.cn](mailto:songcp@henu.edu.cn) (Chunpeng Song) [↑](#footnote-ref-1)
